# TM4SF18 is aberrantly expressed in pancreatic cancer and regulates cell growth

**DOI:** 10.1371/journal.pone.0211711

**Published:** 2019-03-21

**Authors:** Megha Singhal, Mahsa Khatibeghdami, Daniel R. Principe, Georgina E. Mancinelli, Kyle M. Schachtschneider, Lawrence B. Schook, Paul J. Grippo, Sam R. Grimaldo

**Affiliations:** 1 Department of Medicine, Division of Gastroenterology and Hepatology, University of Illinois, Chicago, Illinois, United States of America; 2 University of Illinois College of Medicine, Chicago, Illinois, United States of America; 3 Department of Radiology, University of Illinois at Chicago, Chicago, Illinois, United States of America; 4 Department of Biochemistry and Molecular Genetics, University of Illinois at Chicago, Chicago, Illinois, United States of America; 5 National Center for Supercomputing Applications, University of Illinois at Urbana-Champaign, Urbana, Illinois, United States of America; 6 Department of Animal Sciences, University of Illinois at Urbana-Champaign, Urbana, Illinois, United States of America; University of South Alabama Mitchell Cancer Institute, UNITED STATES

## Abstract

Current therapies for pancreatic ductal adenocarcinoma (PDAC) only modestly impact survival and can be highly toxic. A greater understanding of the molecules regulating this disease is critical for identifying new drug targets and developing more effective therapies. The L6 family of proteins are known to be positive regulators of tumor growth and metastasis among various cancers. However, little is known about the L6 family member TM4SF18. We investigated the expression and localization of the TM4SF18 protein in normal human pancreas and in PDAC tissue. Utilizing immunohistochemistry (IHC) and western blot analysis, our studies for the first time demonstrate that TM4SF18 is highly expressed in PDAC tumor epithelium. Furthermore, we identified TM4SF18 to be expressed in normal acinar tissue and weakly expressed in normal ducts. Although there is minimal expression in normal ducts, we observed increased TM4SF18 levels in preneoplastic ducts and tumor epithelium. To investigate a functional role of TM4SF18 in PDAC we developed stably-expressing inducible shRNA pancreatic cancer cell lines. Knockdown of the TM4SF18 protein led to a significant decrease in Capan-1 cell growth as measured by the MTT assay, demonstrating this molecule to be a novel regulator of PDAC. Uniquely there is no ortholog of the *TM4SF18* gene in mouse or rat prompting us to seek other *in vivo* experimental models. Using IHC and western blot analysis, expression of TM4SF18 was confirmed in the porcine PDAC model, thus we establish an alternative model to investigate this gene. TM4SF18 represents a promising novel biomarker and therapeutic target for pancreatic cancer.

## Introduction

Pancreatic ductal adenocarcinoma cancer (PDAC) is one of the deadliest cancers predicted to be the second-largest cause of cancer-related death by 2020 [1]. Currently the five-year survival rate is at 9% mainly due to poor detection methods and lack of therapeutic options [2]. There is an urgent need to identify new therapeutic and diagnostic markers to help extend patient survival and reduce disease-associated morbidity.

The L6 superfamily consists of six members (TM4SF-1, -4, -5, -18, -19, and -20) characterized to have four-transmembrane domains [3]. Due to structural similarities, members of the L6 family were originally classified as tetraspanins. However, more precise sequence and structural analysis combined with phylogenetic clustering revealed this family as not genuine tetraspanins instead constitute their own family of proteins. Members of L6 superfamily are known to play vital roles in various functions in tumorigenesis including invasion, motility, EMT, adhesion, and cell growth [4–10]. TM4SF1 is one well-studied L6 family member found to be over-expressed in pancreatic cancer and plays a role in pancreatic cancer cell invasion and chemoresistance [10, 11]. TM4SF18 is highly homologous to TM4SF1 but has yet to be studied in any aspect of cancer biology.

Current studies investigated the role of TM4SF18 in PDAC. TM4SF18 was found to be expressed by normal pancreatic acinar cells and weak-to-no expression was seen in the normal ducts. Furthermore, increased expression of this protein was seen in early ductal lesions and tumor cells. RNAi studies showed a regulatory effect of TM4SF18 on cell growth. Finally, we investigated the expression of this protein in the PDAC oncopig model. Our study provides valuable insights into the role of this gene as a novel factor for pancreatic cancer.

## Materials and methods

All animal work was conducted according to relevant national and international guidelines. All animal studies and procedures were approved by The University of Illinois Institutional Animal Care and Use Committee (IACUC; Protocol numbers 11221 for pig).

### Cell lines

Human pancreatic cancer cell lines MIA PaCa-2, Capan-1, PANC-1, BxPC-3, and AsPC-1 were purchased from the American Type Culture Collection (ATCC Manassas, VA). MIA PaCa-2, BxPC-3, AsPC-1, and Panc-1 cells were cultured in Dulbecco's modified Eagle's medium (DMEM) media containing 10% FBS, penicillin (100 units/mL), and streptomycin (100 μg/mL). Capan-1 cells were cultured in Iscove's Modified Dulbecco's Medium (IMDM) containing 10% FBS, penicillin (100 units/mL), and streptomycin (100 μg/mL). Normal Human Pancreatic Ductal Epithelial (HPDE) cells were obtained from Ming-Sound Tsao lab (University Health Network, Canada) [12]. HPDE cells are an immortalized cell-line derived from primary cultures of normal ductal epithelial cells and are grown in Keratinocyte serum-free (KSF) medium supplemented with 50 mg/ml bovine pituitary extract (BPE) and 5 ng/ml epidermal growth factor (EGF). All cells were maintained at 37°C in a humidified incubator with 5% CO_**2**_. Cancer cell-lines have been subjected to authentication by STR profiling and are subjected yearly to mycoplasma screening.

### Human tissue samples

Sections of de-identified formalin-fixed paraffin embedded normal adjacent pancreatic tissues and pancreatic cancer tissues were obtained from the University of Illinois Biorepository following IRB approval. All methods were performed in accordance with the relevant guidelines and regulations. The protocol was approved by the University of Illinois at Chicago Office for the Protection of Research Subjects Institutional Review Board.

### RNAi of TM4SF18

To establish inducible and stable TM4SF18 shRNA in the Capan-1 cell line we used the SMARTvector lentiviral shRNA particles (Dharmacon, Lafayette, CO). Two different shRNA sequences specific for TM4SF18 and one non-targeting control sequence were used. Transduction was done per manufacture instructions. Briefly, cells were transduced at a MOI of 1.0 TU/cell and cell clones expressing respective shRNA were selected with puromycin 5 μg/ml. To induce production of shRNA 1 μg/ml doxycycline was added to growth medium.

### Western blot analysis

Cells were washed with ice-cold PBS and lysed in RIPA buffer (EMD Millipore Burlington, MA) supplemented with phosphatase inhibitors and a protease inhibitor cocktail (Roche, Indianapolis, IN). Cells lysates were incubated for 2 hours at 4°C and centrifuged at 13,000 rpm for 10 min at 4°C to remove cell debris. Protein concentration was determined by Pierce BCA Protein Assay Kit (ThermoFisher Scientific Waltham, MA). Whole cell lysates were loaded on a 12% SDS-polyacrylamide gel and transblotted to nitrocellulose membrane after electrophoretic separation. Blocking was carried out in 5% PBS-milk following which the membrane was incubated with the in-house TM4SF18 antibody overnight (1:500). Our in-house TM4SF18 rabbit polyclonal antibody is directed against the N-terminal region corresponding to the following amino acid sequence GSRKCGGCLSCLLI. The membrane was washed with 1X PBST and probed with HRP-conjugated anti-rabbit antibody (1:1000 dilution) for 1 h followed by ECL (enhanced chemiluminescence, from Bio-Rad, Hercules, CA) detection. For the loading control, membranes were incubated ß-actin antibody for 1 hour at room temp washed and probed with HRP-conjugated anti-mouse antibody.

### Immunohistochemistry and immunofluorescence

For Immunohistochemistry, slides were deparaffinized by xylene and rehydrated by ethanol gradient, heated in a pressure cooker containing antigen unmasking solution from Vector Laboratories (Burlingame, CA). Bloxall solution was used to block the endogenous peroxidases and alkaline phosphatases with a 10-min incubation. Tissues were blocked with 3% BSA in PBST for 2–3 h at room temperature and incubated with TM4SF18 antibody overnight (1:200) at 4°C. Slides were developed with HRP-secondary antibodies, followed by DAB substrate/buffer (Vector laboratories).

For co-immunofluorescence staining, the slides were heated in pressure cooker containing antigen unmasking solution from Vector Laboratories. Tissues were blocked in 3% BSA in PBST for 1–2 h at room temperature followed by overnight incubation at 4°C with primary antibodies against TM4SF18 (1:100) and CK19 (1:100 University of Iowa Hybridoma Bank) or Amylase (1:25 Santa Cruz Dallas, TX), Slides were developed using Alexa flour 488 or 594 conjugated secondary antibodies (1:100, ThermoFisher Scientific) and mounted in DAPI (Vector Laboratories). Slides were imaged using the laser confocal microscope (Zeiss Thornwood, NY).

### MTT assay

Cell viability was assessed using 3-[4,5-dimethylthiazol-2-yl]-3,5-diphenyl tetrazolium bromide (MTT) assay. Briefly, 2000 cells/well were seeded in triplicate in a 96-well plate after incubation with 1 μg/ml doxycycline then MTT (5 mg/ml) was added to the cells containing media and incubated at 37°C for 2 hours. The media were removed, and formazan crystals were solubilized with 200 μl of DMSO. Absorbance at 570 nm was measured using a microplate reader (BioRad, Hercules, CA).

### Wound healing assay-migration

Cells were pre-treated with doxycycline to induce shRNA production then seeded to full density (1.2x10^6^ cells/well) on six-well plates previously coated with collagen-1. Cells were then serum starved for 4 hours and monolayer was scratched twice with a plastic pipette tip. Next, the cells were washed and incubated for up to 36 h in complete media with doxycycline. Micrographs were taken 0h, 24h and 36h at 100x magnification. The percentage of non-recovered wound area was calculated by dividing the non-recovered area after respective time point by the initial wound area at time zero.

### Porcine model of pancreatic cancer

Transgenic LSL-KRAS^G12D^-TP53^R167H^ pigs were generated as previously described [13] and then developed as an inducible PDAC model, as done previously [14]. All experiments involving the use of pigs were approved by the Institutional Animal Care and Use Committee at the University of Illinois. The animals were maintained in a 24h dark light cycle and had access to food and water ad libitum. In brief, animals are dosed with 4x10^9^ PFU of Ad5CMVCre-eGFP directly into the main pancreatic duct to activate Kras^G12D^ and p53^R167H^ mutations in pancreas. For all cases the pancreas was accessed surgically through a ventral midline incision. Anesthesia was induced with TKX (Telazol, Ketamine, Xylazine), intubated and maintained in a surgical plane of anesthesia with Isoflurane. For euthanasia, each animal was sedated and euthanized with sodium pentobarbital overdose (Fatal plus 10 cc/100 lbs.). All animals underwent gross and histopathologic evaluation. Tissue was collected one-year post-induction, formalin fixed, and paraffin embedded.

The Porc-1 cell-line was generated as previously described [14]. In brief, the pancreas from a two-month old female non-induced transgenic pig was collected and immediately cut into 1mm^3^ pieces and digested with Collagenase Ia (1 mg/ml). Cells were then seeded on six well plates and infected with Adeno-CRE at 200 to 500 MOI. The media was changed after six hours and cells maintained at 37°C with 5% CO_2_.

### Statistical analysis

The data were analyzed with unpaired T-test. A *p*-value less than 0.05 was considered statistically significant.

## Results

### TM4SF18 expression in human PDAC

**Fig 1:**
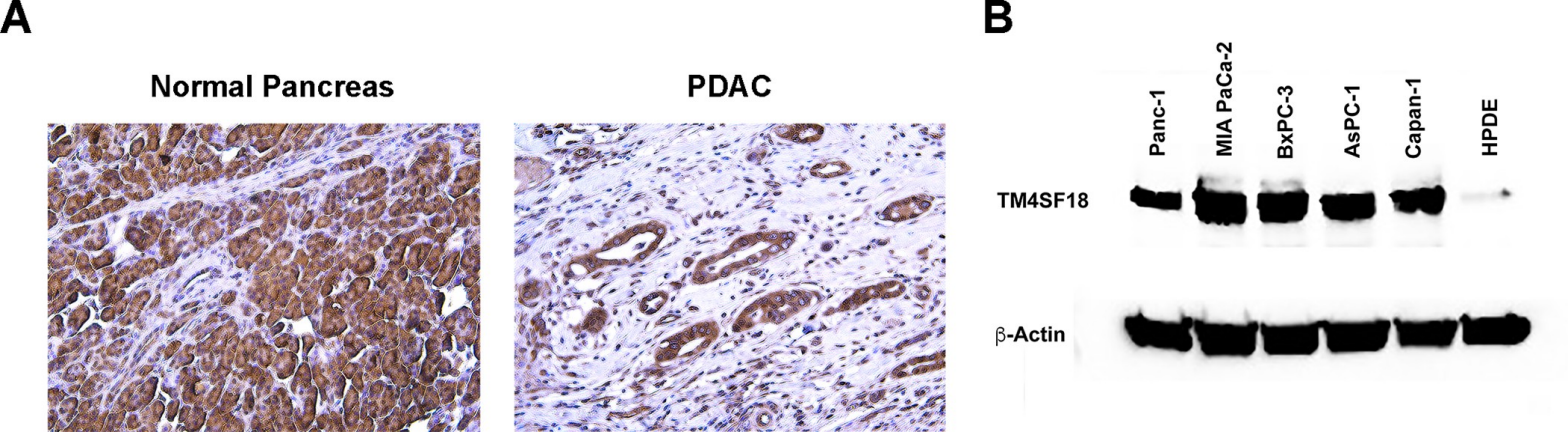
TM4SF18 expression in human pancreatic tissue and cell lines. Immunohistochemical analysis of the TM4SF18 protein showing high expression in normal human exocrine pancreas and within tumor lesions of PDAC tissue (Magnification: x200). B) Western blot analysis of TM4SF18 protein expression in various pancreatic cancer cell lines and a normal pancreatic ductal cell line (HPDE).

We first determined the expression of TM4SF18 in human adjacent normal pancreas and PDAC tissues by immunohistochemical (IHC) analysis (N = 4/group). TM4SF18 exhibited strong staining in both PDAC neoplastic lesions and normal pancreatic acinar tissue (Fig 1A). To validate the specificity of the TM4SF18 antibody we performed a similar staining in the presence of a blocking peptide (S1A Fig). We next assessed TM4SF18 expression in the pancreatic cancer cell lines Panc-1, MiaPaca-2, BxPC-3, AsPC1, and Capan-1, as well as the non-malignant human pancreatic ductal epithelium (HPDE) cell line by western blot analysis. There was a ~20-fold elevated expression of TM4SF18 in the cancer cell lines compared to the normal ductal cell line (Fig 1B). These data demonstrate that TM4SF18 is highly expressed by PDAC tumor cells.

### TM4SF18 localizes to normal acinar tissue and ductal tumor lesions

**Fig 2:**
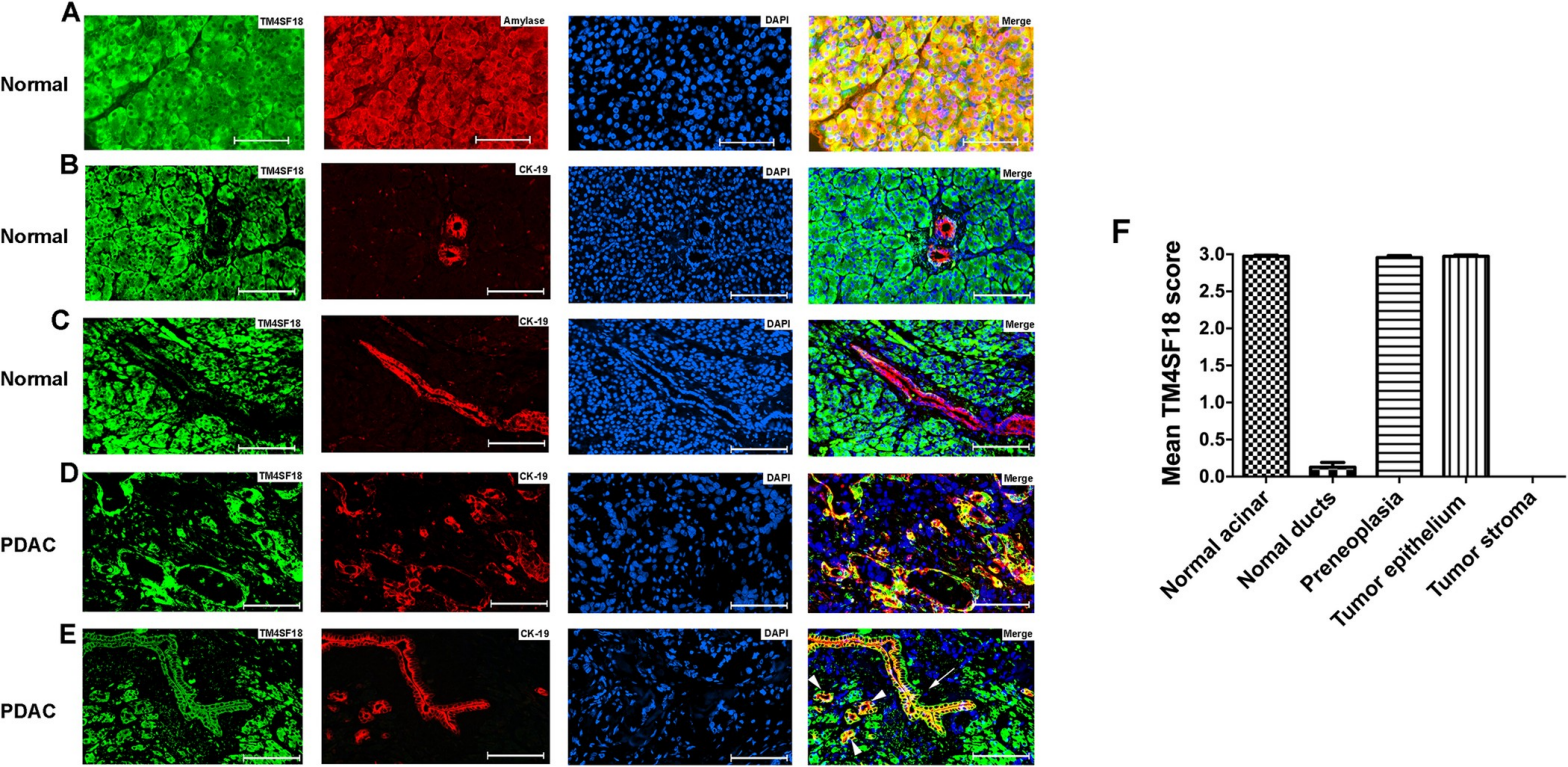
Co-staining of TM4SF18 with pancreatic-specific cell markers. Co-staining of TM4SF18 (green) in normal pancreatic tissue with the pancreatic acinar cell marker amylase (red). B and C) A cross section and a longitudinal section co-stained with TM4SF18 (green) and the ductal marker CK-19 (red) in normal pancreas. D) Co-staining of TM4SF18 with CK-19 in a human PDAC section with advanced tumor growth. E) Co-staining of TM4SF18 with CK-19 in a section with cells undergoing acinar-ductal-metaplasia (arrow head) and a PanIN lesion (arrow). F) Mean TM4SF18 expression was calculated by scoring the images from 0–3, figures are representative of at least 3 patients. Scale bar = 100 μm, Magnification: x200.

To better understand the expression pattern of TM4SF18 in both normal and diseased pancreatic tissue, we performed co-immunofluorescence staining of TM4SF18 with known pancreatic cell markers. In normal pancreas TM4SF18 co-localized predominantly with the acinar cell marker, amylase (Fig 2A). Furthermore, TM4SF18 showed weak-to-no staining in normal ducts when co-stained with CK-19, a marker of normal and pathologic ductal cells (Fig 2B and 2C). In contrast, TM4SF18 showed strong co-localization with the CK-19+ tumor epithelium (Fig 2D). In addition, TM4SF18 was found to be expressed in pre-neoplastic lesions including ADM (acinar-to-ductal metaplasia) and a PanIN (pancreatic interepithelial neoplasia) lesions (Fig 2E). Expression of each cell-type was scored, and average calculated (Fig 2F). The staining was also done with a no primary antibody control to validate the specificity of the TM4SF18 antibody for immunofluorescence (S1B Fig). These results indicate that TM4SF18 is an acinar-specific protein that also is elevated in abnormal ducts and tumor cells.

### RNAi-mediated silencing of TM4SF18 expression

**Fig 3:**
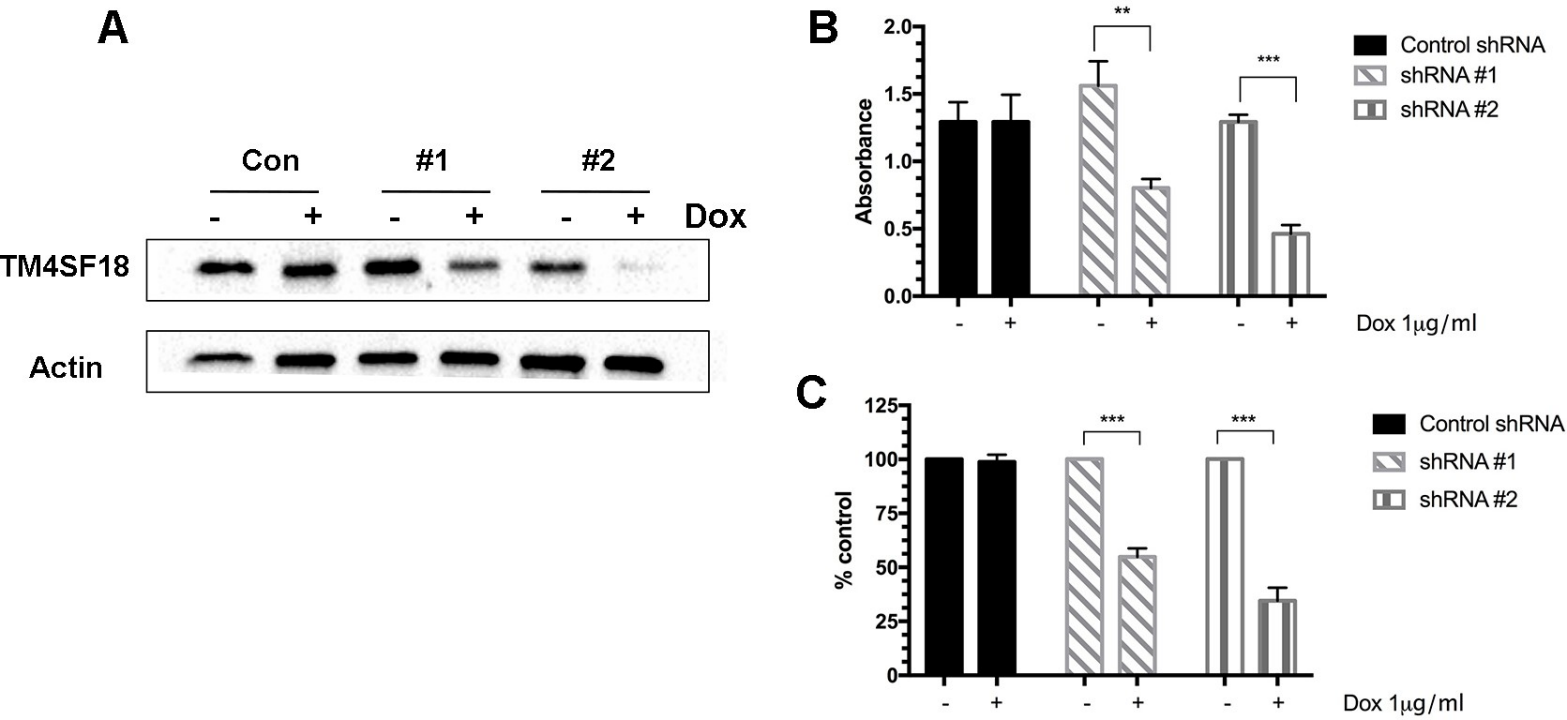
TM4SF18-silencing decreases pancreatic cancer cell growth. Western blot analysis of three stable Capan-1 cell lines containing either TM4SF18 or control shRNA treated with or without doxycycline treatment. B) Colorimetric MTT assay showing a decrease in cell viability in response to five days post-induction of TM4SF18 shRNA or control shRNA. C) Number of viable cells following five days post dox-induction of TM4SF18 or control shRNA. **p<0.01, ***p<0.001.

To identify a role of TM4SF18 in pancreatic cancer cell biology we utilized doxycycline-inducible shRNA constructs to knockdown TM4SF18 in Capan-1 cells. We established stable cell lines with two different TM4SF18 shRNA sequences and a control shRNA. In response to seven days of 1 μg/ml doxycycline (dox) treatment, TM4SF18 shRNA #1 showed an approximately 60% decrease in protein levels while shRNA #2 revealed a more efficient silencing with a >90% decrease in protein levels (Fig 3A). We next investigated the effect PDAC cell growth upon silencing of TM4SF18 with two independent assays. TM4SF18 shRNA#1 and shRNA#2 showed decrease in cell growth by 48% and 64%, respectively, when compared to non-induced cells (Fig 3B) in an MTT assay. These results were further affirmed by counting viable cells following dox-induction showing a 45% and 66% decrease in number of cells with shRNA#1 and shRNA#2, respectively, when compared to non-induced cells (Fig 3C). These results indicate that TM4SF18 promotes pancreatic cancer cell growth.

### TM4SF18-silencing does not modulate Capan-1 cell migration

**Fig 4:**
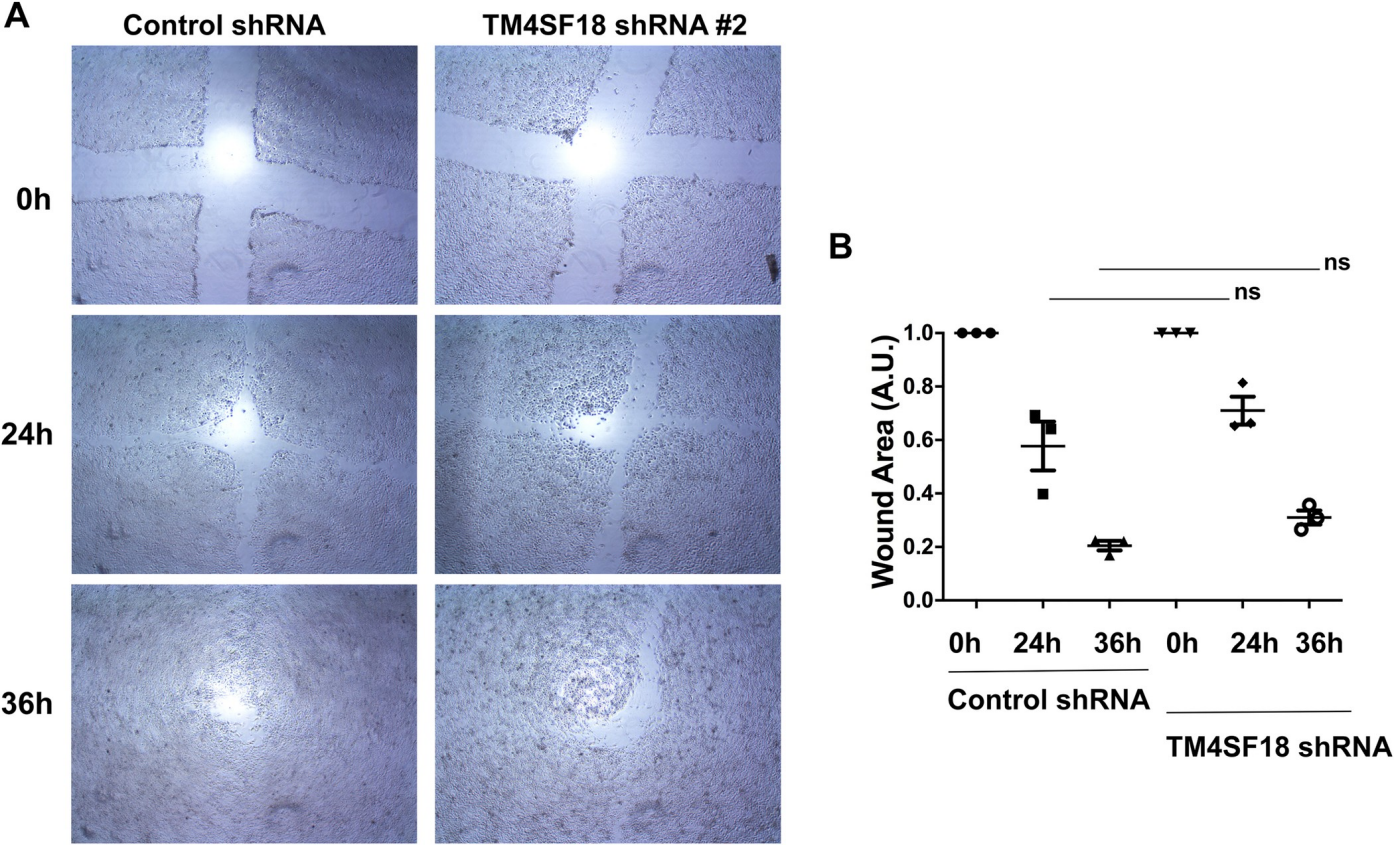
TM4SF18-silencing does not affect migration. A) Phase/contrast photomicrographs of induced control shRNA and TM4SF18 shRNA cell-lines following a wound opening at time 0 h, 24 h, and 36 h. B) Percentage of the wound closure at each timepoint.

The L6 members are known to have a role in cancer cell migration [7, 10, 15, 16] and thus we investigated if TM4SF18 drives pancreatic cancer cell migration. We performed a wound healing assay on confluent TM4SF18 shRNA #2 and control shRNA stable cell lines that had been pre-treated with doxycycline (Fig 4). Silencing of TM4SF18 did not significantly affect the migration of cells as compared to control Capan-1 cells suggesting that TM4SF18 is not involved in pancreatic cancer cell migration.

### TM4SF18 is expressed in the PDAC oncopig model

**Fig 5:**
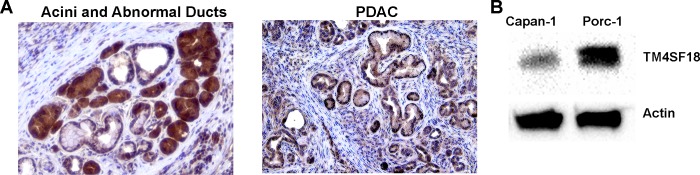
Expression of TM4SF18 in the PDAC oncopig model. A) Immunohistochemical staining of TM4SF18 in acinar cells, abnormal ducts, and tumor epithelium in a transgenic pig (LSL-Kras^G12D^ and LSL-p53^R167H^). B) TM4SF18 western blot analysis of the Porc-1 cell-line.

TM4SF18 is a promising new molecule that warrants further studies in PDAC biology. Since there is no mouse or rat ortholog of the *TM4SF18* gene, we are limited in the use of these animal model systems. However, TM4SF18 is predicted to be expressed in the domestic pig (sus scrofa; accession #XM_003132479.4) Our group has recently developed an oncopig PDAC model system [14]. We therefore assessed the expression of TM4SF18 in the oncopig model by IHC of pancreatic tissue and by western blot analysis of a primary cell-line derived from this model. Consistent with our previous findings, in human tissue, TM4SF18 was expressed by normal acini, ADM, and late PDAC lesions (Fig 5A). In addition, western blot analysis of the Porc-1 cell line showed a robust signal for TM4SF18 (Fig 5B). These results confirm that the porcine PDAC model express the TM4SF18 protein and therefore is a suitable platform for future investigations of this gene.

## Discussion

In summary, to our knowledge we provide the first evidence that TM4SF18 is expressed in normal and pancreatic cancer tissue including acinar tissue, pre-neoplastic ductal lesions, and tumor epithelium. In addition, we define a regulatory role of TM4SF18 in pancreatic cancer cell growth. Lastly, we identified the porcine PDAC model as a platform for future investigations into this promising new target for pancreatic cancer.

The L6 family of proteins continue to gain interest in cancer research due to their multiple roles in tumorigenesis. TM4SF1 is a notable family member shown to regulate cell proliferation, adhesion, and metastasis in various cancers such as lung cancer [17], prostate cancer [4], breast cancer [7], liver cancer [18, 19], bladder cancer [20], and pancreatic cancer [5, 10, 21]. In pancreatic cancer, TM4SF1 has been shown to promote metastasis and cell motility be inducing invadopodia and regulating MMP activity [21]. Although TM4SF1 and TM4SF18 share ~60% amino acid sequence homology they appear to have distinct roles in pancreas biology. In normal pancreas, TM4SF1 is only weakly expressed by normal ducts [6, 10] in contrast to the high TM4SF18 expression in normal acinar tissue. Furthermore, the role of TM4SF1 in PDAC appears to be restricted to migration and invasion but does not regulate cell growth [10], in contrast we identified TM4SF18 to have a significant role to regulate PDAC cell growth but does not modulate migration. TM4SF5 is another L6 family member that is up-regulated in PDAC, but its functional role is unknown [22]. To date, our data shows that TM4SF18 is the only L6 member known to promote PDAC cell growth. It will be important to further define the role of TM4SF18 in PDAC biology and understand its interplay with other members of the L6 family to fully encompass the impact of these factors in pancreas biology.

A key event during early PDAC progression is the transdifferentiation of acinar cells into ductal cells known as acinar-to-ductal metaplasia [23]. Typically, during ADM cells undergo loss of acinar-specific gene expression including Nr5a2, Mist1, amylase, and Ptf1a and there is an upregulation of ductal markers such as Sox9, Hnf6, and CK-19. Here we show that TM4SF18 appears to be a unique acinar-specific protein molecule that does not become reduced during PDAC progression but rather is increased during ductal cell differentiation. It is unclear whether TM4SF18 is a factor involved in the transdifferentiation process or possibly co-opted to promote tumor cell growth. Future investigations of TM4SF18 may help to gain a better understanding of PDAC initiation and development.

The *TM4SF18* gene is not present in several members of the muridae and cricetidae rodent families including mice and rats. Although the *TM4SF18* ortholog is retained in several other rodents including the thirteen-line ground squirrel, guinea pig, and naked mole rat. Thus, the *TM4SF18* gene represents a unique evolutionary break among certain families and a lineage-specific loss in certain rodents. The lack of an ortholog in hallmark experimental models poses a difficult task for future investigations. Xenograft tumor models will be important to further define the role of TM4SF18 in cancer biology. However, for a better understanding in developmental biology and site-specific TM4SF18 expression the oncopig model may be needed. The oncopig model has the added benefit of being a more accurate representation to human physiology in particular to pancreas anatomy and immunology and thus the potential to be a more powerful PDAC model when compared to rodent approaches [14, 24–25].

In conclusion, our studies show a promising new molecule that may serve as a biomarker for early and late PDAC disease. Furthermore, since L6 family members are characterized as cell-surface molecules having two extracellular loop domains [3], projects TM4SF18 a feasible target for future drug development.

## Supporting information

S1 Fig Validation of the N-Terminal TM4SF18 antibody. A) IHC analysis of adjacent normal human pancreas incubated with N-Terminal antibody alone or pre-incubated with 10-fold excess peptide. B) Immunofluorescence staining of adjacent normal human pancreas with or without the N-terminal antibody.
(TIFF)

S1 File Minimal data set. Values and raw numbers used for the figures.
(PDF)
