# Supplementary figures and images for: TM4SF18 is aberrantly expressed in pancreatic cancer and regulates cell growth
Source: PLoS One. 2019 Mar 21;14(3):e0211711. doi: 10.1371/journal.pone.0211711 (PMC6428261; doi:10.1371/journal.pone.0211711)

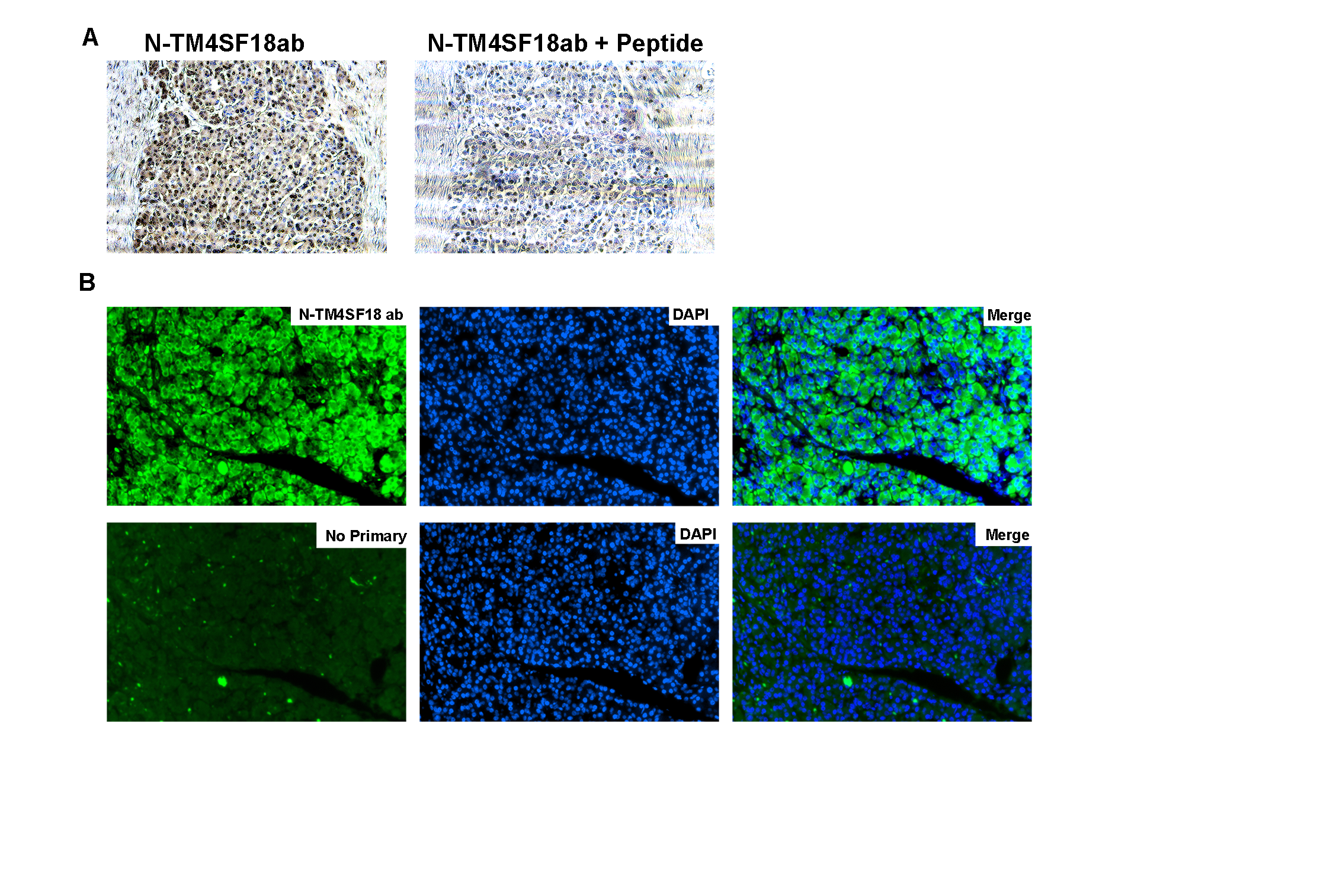

Supplement: S1 Fig — A) IHC analysis of adjacent normal human pancreas incubated with N-Terminal antibody alone or pre-incubated with 10-fold excess peptide. B) Immunofluorescence staining of adjacent normal human pancreas with or without the N-terminal antibody. (TIFF) [file pone.0211711.s001.tiff]
