# Supplementary material for: TM4SF18 is aberrantly expressed in pancreatic cancer and regulates cell growth
Source: PLoS One. 2019 Mar 21;14(3):e0211711. doi: 10.1371/journal.pone.0211711 (PMC6428261; doi:10.1371/journal.pone.0211711)
Supplement: S1 File — Values and raw numbers used for the figures. (PDF) [file pone.0211711.s002.pdf]

Fig 2F. Scoring of TM4SF18 intensity in each compartment from 0-3:

Normal acinar	Normal ducts	Preneoplasia	Tumor epithelium	Tumor stroma
3.00	0.20	3.00	3.00	0
2.95	0.00	2.96	2.98	0
2.97	0.18	2.91	2.94	0

Fig 3B. Absorbance data from MTT assay

Group	Abs-1	Abs-2	Abs-3	Mean	SD	N
Control -Dox	1.42061364	1.13331364	1.32571364	1.29321364	0.14638139	3
Control +Dox	1.18291364	1.16881364	1.52491364	1.29221364	0.20164739	3
shRNA-1 -Dox	1.39301364	1.53801364	1.75211364	1.56104697	0.18065465	3
shRNA-1 +Dox	0.87851364	0.75611364	0.77391364	0.80284697	0.06613088	3
shRNA-2 -Dox	1.35411364	1.25221364	1.27031364	1.29221364	0.05436552	3
shRNA-2 +Dox	0.43261364	0.41941364	0.53701364	0.46301364	0.06442484	3

Fig 3C. Percent of control of cell count assay

Group	#Cells/well-1	#Cells/well-2	#Cells/well-3	Mean	% of -Dox sample	SD	N
Control -Dox	180000	176000	197000	184333.3	100	0	3
Control +Dox	176000	169000	202000	182333.3	98.76	3.35	3
shRNA-1 -Dox	105000	122000	113000	113333.3	100	0	3
shRNA-1 +Dox	57000	72000	58000	62333.33	54.7	4.04	3
shRNA-2 -Dox	229000	202000	198000	209666.7	100	0	3
shRNA-2 +Dox	67000	67000	82000	72000	34.4	6.11	3

Fig 4B. Migration assay: Open area measured in pixel distance via imagej software. Raw data and percent of control

Group	Well-1	Well-2	Well-3	% of control-1	% of control-2	% of control-3
Control 0h	547	502	553	1	1	1
Control 24hour	379	322	220	0.69287	0.641434	0.39783
Control 36hr	121	85	124	0.221207	0.169323	0.224231
siRNA#2 0h	490	398	448	1	1	1
siRNA #2 24h	320	324	297	0.653061	0.81407	0.662946
siRNA #2 36h	151	142	119	0.308163	0.356784	0.265625
